# The Italian investment in telemedicine: a breakthrough model of care in primary care

**DOI:** 10.1093/eurpub/ckac129.420

**Published:** 2022-10-25

**Authors:** A Borghini, D Mantoan, S Paone, I Leta, G Siccardi

**Affiliations:** National Agency for Regional Health Services, AGENAS, Rome, Italy

## Abstract

**Issue/Problem:**

Aging populations, worsening burden of chronic disease and recent pandemic has accelerated awareness and the importance of telemedicine in providing continuity of healthcare.

**Description of the problem:**

AGENAS is the public body responsible for the implementation of telemedicine investment (€1 billion) in the context of the NextGenerationEU plan. AGENAS has built up a working group expert panel to define the technical and informatics features of the investment. The project consists of the realization of the national telemedicine platform and the regional telemedicine services. Italian regions will implement telemedicine services based on the national guidelines defined by AGENAS, that will also monitor it through key performance indicators outlined on the basis of best practices and scientific evidence of multidimensional evaluation.

**Results:**

National telemedicine platform will improve, optimise and standardise telemedicine services throughout the Country, considering what may already be available in regional and local healthcare contexts. Regarding telemedicine services in regional context, that will be implemented within the NextGenerationEU, they will be focused on the telemonitoring of high prevalence conditions (i.e. cardiological, respiratory, diabetes, neurological and oncological) as well as other services such as televisit, teleconsultation and teleassistance. Connecting patient's home with healthcare system provide benefits for patients and their families, who will be able to interact with healthcare professionals, obtaining consultation and monitoring of their health.

**Lessons:**

The implementation of the investment, aiming at improving equity and integration of care, will contribute to provide real world evidence about usage, benefits and potential risk of the telemedicine in primary care for the management of chronic diseases.

**Key messages:**

• The investment under the Next Generation EU plan it is the lifetime chance to transform Italian healthcare service and draw a new framework to cope with the high demand in telemedicine.

• Improving telemedicine services will determine a breakthrough in management of patient with chronic diseases in the Italian primary care sector.

